# Scheduling Jobs with Variable Job Processing Times on Unrelated Parallel Machines

**DOI:** 10.1155/2014/242107

**Published:** 2014-05-26

**Authors:** Guang-Qian Zhang, Jian-Jun Wang, Ya-Jing Liu

**Affiliations:** Faculty of Management and Economics, Dalian University of Technology, Dalian 116024, China

## Abstract

*m* unrelated parallel machines scheduling problems with variable job processing times are considered, where the processing time of a job is a function of its position in a sequence, its starting time, and its resource allocation. The objective is to determine the optimal resource allocation and the optimal schedule to minimize a total cost function that dependents on the total completion (waiting) time, the total machine load, the total absolute differences in completion (waiting) times on all machines, and total resource cost. If the number of machines is a given constant number, we propose a polynomial time algorithm to solve the problem.

## 1. Introduction

In classical scheduling theory, it is assumed that the job processing times are fixed and constant values. In practice, however, we often encounter settings in which job processing times may be subject to change due to the deterioration effects and/or learning effects and/or controllable processing times. Extensive surveys of different scheduling models and problems involving deteriorating jobs can be found in Alidaee and Womer [1], Cheng et al. [2], and Gawiejnowicz [3]. A recent survey of the scheduling problems with learning effects could be found in Biskup [4]. A recent survey of the scheduling problems with controllable processing times was given by Shabtay and Steiner [5]. More recent papers that have considered scheduling problems with deterioration effects and/or learning effects and/or controllable processing times include Bai et al. [6, 7], Cheng et al. [8], Gorczyca and Janiak [9], Hsu and Yang [10], Huang and Wang [11], Lee et al. [12], Leyvand et al. [13], Nian and Mao [14], Shabtay and Steiner [15], Wang et al. [16], J. B. Wang and M. Z. Wang [17], J. B. Wang and J. J. Wang [18], Wang et al. [19–21], Wei et al. [22], Wu and Lee [23], Wu and Liu [24], Yang and Kuo [25], S. J. Yang and D. L. Yang [26], Yang et al. [27], Yin et al. [28–31], and Zhao and Tang [32]. Mosheiov and Sidney [33] considered the single machine model:

(1) $$\begin{array}{c} p_{j}=a_{j}r^{b_{j}}, \end{array}$$

where *a*_*j*_ is the original (normal) processing time of job *J*_*j*_, *p*_*j*_ is the actual processing time of job *J*_*j*_, *r* is the position of job *J*_*j*_ when scheduled on the machine, and *b*_*j*_ ≤ 0 is the job-dependent learning index of job *J*_*j*_. Yang and Kuo [25] considered single machine scheduling with the deterioration and learning effect in which the job processing times are

(2) $$\begin{array}{c} p_{j}=a_{j}r^{b}+\alpha t, \end{array}$$

where *t* is the starting time of job *J*_*j*_, *b* ≤ 0 is a learning index, and *α* ≥ 0 is a common deterioration rate for all the jobs. Shabtay and Steiner [15] considered single machine scheduling with the resource allocation model in which the job processing times are

(3) $$\begin{array}{c} p_{j}=a_{j}-\beta_{j}u_{j}, \end{array}$$

where *u*_*j*_ is the amount of a nonrenewable resource allocated to job *J*_*j*_, with $0\leq u_{j}\leq {\overset{-}{u}}_{j}<a_{j}/\beta_{j}$, where ${\overset{-}{u}}_{j}$ denote the maximum amount of resource allocated to job *J*_*j*_ and *β*_*j*_ is the positive compression rate of job *J*_*j*_.

Wang et al. [19] considered single machine scheduling problem where the job processing time is a function of position in a sequence, starting time, and resource allocation to this job on a single machine; that is, the model

(4) $$\begin{array}{c} p_{j}=a_{j}r^{b}+\alpha t-\beta_{j}u_{j}, \end{array}$$

where $0\leq u_{j}\leq {\overset{-}{u}}_{j}<a_{j}n^{b}/\beta_{j}$. For minimizing a cost function containing makespan, total completion (waiting) time, total absolute differences in completion (waiting) times, and total resource cost, they proved that the problem can be solved in polynomial time.

Wang et al. [19] considered single machine scheduling problem with effect of deterioration, learning, and resource allocation simultaneously. The example of the phenomena of deterioration, learning effect, and resource allocation occurring simultaneously can be found in Wang et al. [19]. However, the parallel machine scheduling is interesting and closer to real problems in practice (Cheng et al. [8], Hsu and Yang [10], Huang and Wang [11], Yang et al. [27], and Yin et al. [30]). This paper extends the single machine scheduling results of Wang et al. [19], by considering unrelated parallel machine scheduling problems that include the one given in Wang et al. [19] as a special case. The remainder of this paper is organized as follows. In Section 2 we formulate the model. In Sections 3 and 4, we show that the problem can be solved in polynomial time for two different cost functions. The last section contains some conclusions.

## 2. Problem Formulation

The model is described as follows. There are *n* independent jobs {*J*_1_, *J*_2_,…, *J*_*n*_} to be processed on *m* unrelated parallel machines {*M*_1_, *M*_2_,…, *M*_*m*_}. Each of them is available at time 0. The machine can handle one job at a time, and preemption is not allowed. Let *n*_*i*_ denote the number of jobs assigned to *M*_*i*_  (*i* = 1,2,…, *m*) and *P*(*n*, *m*) = (*n*_1_, *n*_2_,…, *n*_*m*_) denote a job-allocation vector, where (*n*_1_ + *n*_2_ + ⋯*n*_*m*_ = *n*). We assume, as in most practical situations, that *m* < *n* and *m* are a given constant. Each job can be processed on any one of the *m* unrelated parallel machines. Associated with each job *J*_*j*_  (*j* = 1,2,…, *n*) on machine *M*_*i*_, there is a normal processing time *a*_*ij*_. Let *p*_*ij*_ denote the actual processing time for job *J*_*j*_ on machine *M*_*i*_. In this paper, we consider a general unrelated parallel machines model stemming from Yang and Kuo [25], Shabtay and Steiner [15], and Wang et al. [19]; that is,

(5) $$\begin{array}{c} p_{ij}=a_{ij}r^{b_{ij}}+\alpha t-\theta_{ij}u_{ij}, \end{array}$$

where *r* is the position of job *J*_*j*_ when scheduled on machine *M*_*i*_, *t* is the starting time of job *J*_*j*_ on machine *M*_*i*_, *b*_*ij*_ ≤ 0 is a job-dependent learning index, *α* ≥ 0 is a common deterioration rate for all the jobs, *θ*_*ij*_ ≥ 0 is the positive compression rate of job *J*_*j*_ on machine *M*_*i*_, and *u*_*ij*_ is the amount of resource that can be allocated to job *J*_*j*_ on machine *M*_*i*_, with $0\leq u_{ij}\leq {\overset{-}{u}}_{ij}<a_{ij}n^{b_{ij}}/\theta_{ij}$, where ${\overset{-}{u}}_{ij}$ is the upper bound on the amount of resource that can be allocated to job *J*_*j*_ on machine *M*_*i*_.

Let *J*_*i*[*j*]_ denote the *j*th job on machine *M*_*i*_ and *C*_*i*[*j*]_(*W*_*i*[*j*]_) denote the completion (waiting) time of job *J*_*i*[*j*]_; *p*_*i*[*j*]_, *a*_*i*[*j*]_, *b*_*i*[*j*]_, *θ*_*i*[*j*]_, *u*_*i*[*j*]_, and ${\overset{-}{u}}_{i[j]}$ are defined similarly, where *W*_*i*[*j*]_ = *C*_*i*[*j*]_ − *p*_*i*[*j*]_. Let *L*_*i*_ = max⁡{*C*_*ij*_ | *j* = 1,2,…, *n*_*i*_}, *TC*_*i*_ = ∑_*j*=1_^*n*_*i*_^ *C*_*ij*_ (*TW*_*i*_ = ∑_*j*=1_^*n*_*i*_^ *W*_*ij*_), and *TADC*_*i*_ = ∑_*j*=1_^*n*_*i*_^∑_*l*=*j*_^*n*_*i*_^ | *C*_*ij*_ − *C*_*il*_| (*TADW*_*i*_ = ∑_*j*=1_^*n*_*i*_^∑_*l*=*j*_^*n*_*i*_^ | *W*_*ij*_ − *W*_*il*_|) be the load, the total completion (waiting) times, and the total absolute differences in completion (waiting) times of machine *M*_*i*_. Then, the total machine load, the total completion (waiting) time, and the total absolute deviation of job completion (waiting) time on all machines are ∑_*i*=1_^*m*^ *L*_*i*_, ∑_*i*=1_^*m*^ *TC*_*i*_ (∑_*i*=1_^*m*^ *TW*_*i*_), and ∑_*i*=1_^*m*^ *TADC*_*i*_ (∑_*i*=1_^*m*^ *TADW*_*i*_), respectively. The objective is to determine the optimal resource allocations and the optimal schedule on the machines so that the corresponding value of the following cost functions is optimal:

(6) Z1(π,u)=α1∑i=1mLi+α2∑i=1mTCi+α3∑i=1mTADCi +α4∑i=1m ∑j=1niGijuij,

(7) Z2(π,u)=α1∑i=1mLi+α2∑i=1mTWi+α3∑i=1mTADWi +α4∑i=1m ∑j=1niGijuij,

where weights *α*_1_ ≥ 0, *α*_2_ ≥ 0, *α*_3_ ≥ 0, and *α*_4_ ≥ 0 are given constants and *G*_*ij*_ is the per time unit cost associated with the resource allocation. Using the three-field notation introduced by Graham et al. [34], the corresponding scheduling problem is denoted by *Rm* | *p*_*ij*_ = *a*_*ij*_ *r* ^*b*_*ij*_^ + *αt* − *θ*_*ij*_ *u*_*ij*_ | *α*_1_∑_*i*=1_^*m*^ *L*_*i*_ + *α*_2_∑_*i*=1_^*m*^ *TC*_*i*_ + *α*_3_∑_*i*=1_^*m*^ *TADC*_*i*_ + *α*_4_∑_*i*=1_^*m*^∑_*j*=1_^*n*_*i*_^ *G*_*ij*_ *u*_*ij*_ and *Rm* | *p*_*ij*_ = *a*_*ij*_ *r* ^*b*_*ij*_^ + *αt* − *θ*_*ij*_ *u*_*ij*_ | *α*_1_∑_*i*=1_^*m*^ *L*_*i*_ + *α*_2_∑_*i*=1_^*m*^ *TW*_*i*_ + *α*_3_∑_*i*=1_^*m*^ *TADW*_*i*_ + *α*_4_∑_*i*=1_^*m*^∑_*j*=1_^*n*_*i*_^ *G*_*ij*_ *u*_*ij*_.

## 3. Problem *Rm* | *p*_*ij*_ = *a*_*ij*_ *r* ^*b*_*ij*_^ + *αt* − *θ*_*ij*_ *u*_*ij*_ | *α*_1_∑_*i*=1_^*m*^ *L*_*i*_ + *α*_2_∑_*i*=1_^*m*^ *TC*_*i*_ + *α*_3_∑_*i*=1_^*m*^ *TADC*_*i*_ + *α*_4_∑_*i*=1_^*m*^∑_*j*=1_^*n*_*i*_^ *G*_*ij*_ *u*_*ij*_

If the number of jobs on machine *M*_*i*_ is known in advance, from Wang et al. [19], we have

(8) Ci[j]=∑l=1j(1+α)j−l(ai[l]lbi[l]−βi[l]ui[l]),pi[j]=ai[j]jbi[j]−βi[j]ui[j]+α∑l=1j−1(1+α)j−1−l(ai[l]lbi[l]−βi[l]ui[l]).

From Kanet [35], we have *TADC*_*i*_ = ∑_*j*=1_^*n*_*i*_^(*j* − 1)(*n*_*i*_ − *j* + 1)*p*_*i*[*j*]_; in addition, *C*_*i*[*j*]_ = ∑_*l*=1_^*j*^ *p*_*i*[*l*]_, *L*_*i*_ = ∑_*j*=1_^*n*_*i*_^ *p*_*i*[*j*]_, and *TC*_*i*_ = ∑_*j*=1_^*n*_*i*_^ *C*_*i*[*j*]_ = ∑_*j*=1_^*n*_*i*_^(*n*_*i*_ − *j* + 1)*p*_*i*[*j*]_; hence, we have

(9) Z1(π,u)=α1∑i=1m ∑j=1nipi[j]+α2∑i=1m ∑j=1ni(ni−j+1)pi[j] +α3∑i=1m ‍∑j=1ni(j−1)(ni−j+1)pi[j] +α4∑i=1m ∑j=1niGi[j]ui[j]=∑i=1m ∑j=1ni[α1+α2(ni+1−j)+α3(j−1)(ni−j+1)]pi[j] +α4∑i=1m ∑j=1niGi[j]ui[j]=∑i=1m ∑j=1niωijpi[j]+α4∑i=1m ∑j=1niGi[j]ui[j]=∑i=1m ∑j=1niωij(ai[j]jbi[j]−βi[j]ui[j]+α∑l=1j−1(1+α)j−1−l(ai[l]lbi[l]−βi[l]ui[l])) +α4∑i=1m ∑j=1niGi[j]ui[j]=∑i=1m[ωi1(ai[1]1bi[1]−βi[1]ui[1])+ωi2(ai[2]2bi[2]−βi[2]ui[2]+α(ai[1]1bi[1]−βi[1]ui[1]))+ωi3(ai[3]3bi[3]−βi[3]ui[3]+α(ai[2]2bi[2]−βi[2]ui[2]+(1+α)×(ai[1]1bi[1]−βi[1]ui[1])))+ωi4(ai[4]4bi[4]−βi[4]ui[4]+α(ai[3]3bi[3]−βi[3]ui[3]+(1+α) ×(ai[2]2bi[2]−βi[2]ui[2]) + (1+α)2(ai[1]1bi[1]−βi[1]ui[1])))+⋯+ωi,ni−1(ai[ni−1](ni−1)bi[ni−1]−βi[ni−1]ui[ni−1]+α(ai[ni−2](ni−2)bi[ni−2] −βi[ni−2]ui[ni−2]+(1+α)× (ai[ni−3](ni−3)bi[ni−3] −βi[ni−3]ui[ni−3])+⋯+(1+α)ni−4(ai[2]2bi[2]−βi[2]ui[2])+(1+α)ni−3(ai[1]1bi[1]−βi[1]ui[1])))+ωini(ai[ni]nibi[ni]−βi[ni]ui[ni]+α(ai[ni−1](ni−1)bi[ni−1]−βi[ni−1]ui[ni−1]+(1+α)(ai[ni−2](ni−2)bi[ni−2]−βi[ni−2]ui[ni−2])+⋯+(1+α)ni−3(ai[2]2bi[2]−βi[2]ui[2])+(1+α)ni−2(ai[1]1bi[1]−βi[1]ui[1])))] +α4∑i=1m ∑j=1niGi[j]ui[j]=∑i=1m[(ωi1+αωi2+α(1+α)ωi3+⋯+α(1+α)n−2ωini)×(ai[1]1bi[1]−βi[1]ui[1])+(ωi2+αωi3+α(1+α)ωi4+⋯+α(1+α)ni−3ωini)(ai[2]2bi[2]−βi[2]ui[2])+(ωi3+αωi4+α(1+α)ωi5+⋯+α(1+α)ni−4ωini)(ai[3]3bi[3]−βi[3]ui[3])+⋯+(ωi,ni−1+αωini)(ai[ni−1]−βi[ni−1]ui[ni−1])+ωini(ai[ni]nibi[ni]−βi[ni]ui[ni])] +α4∑i=1m ∑j=1niGi[j]ui[j]=∑i=1m ∑j=1niΩijai[j]jbi[j]+∑i=1m ∑j=1ni(α4Gi[j]−βi[j]Ωij)ui[j],

where *ω*_*ij*_ = *α*_1_ + *α*_2_(*n*_*i*_ + 1 − *j*) + *α*_3_(*j* − 1)(*n*_*i*_ − *j* + 1) and

(10)   Ωi1=ωi1+αωi2+α(1+α)ωi3+⋯+α(1+α)ni−2ωini,  Ωi2=ωi2+αωi3+α(1+α)ωi4+⋯+α(1+α)ni−3ωini,  Ωi3=ωi3+αωi4+α(1+α)ωi5+⋯+α(1+α)ni−4ωini,     ⋮ Ωi,ni−1=ωi,ni−1+αωini,  Ωini=ωini.

From (9), for any given sequence on all machines, the optimal resource allocation on all machines can be obtained by the following.

**Lemma 1**

For a given sequence, the optimal resource allocation of the problem *Rm* | *p*_*ij*_ = *a*_*ij*_ *r* ^*b*_*ij*_^ + *αt* − *θ*_*ij*_ *u*_*ij*_ | *α*_1_∑_*i*=1_^*m*^ *L*_*i*_ + *α*_2_∑_*i*=1_^*m*^ *TC*_*i*_ + *α*_3_∑_*i*=1_^*m*^ *TADC*_*i*_ + *α*_4_∑_*i*=1_^*m*^∑_*j*=1_^*n*_*i*_^ *G*_*ij*_ *u*_*ij*_ can be determined by

(11) $$\begin{array}{c} u_{i[j]}^{*}=\{\begin{array}{cc} {\overset{-}{u}}_{i[j]}, & if\;\;\alpha_{4}G_{i[j]}-\beta_{i[j]}\Omega_{ij}<0,\\ u_{i[j]}\in \left[0,{\overset{-}{u}}_{i[j]}\right], & if\;\;\alpha_{4}G_{i[j]}-\beta_{i[j]}\Omega_{ij}=0,\\ 0, & if\;\;\alpha_{4}G_{i[j]}-\beta_{i[j]}\Omega_{ij}>0, \end{array} \end{array}$$

where *u*_*i*[*j*]_*, *i* = 1,2,…, *m*; *j* = 1,2,…, *n*_*i*_, represents the optimal resource allocation of the job in position *j* on machine *M*_*i*_.

**Proof**

For the problem *Rm* | *p*_*ij*_ = *a*_*ij*_ *r* ^*b*_*ij*_^ + *αt* − *θ*_*ij*_ *u*_*ij*_ | *α*_1_∑_*i*=1_^*m*^ *L*_*i*_ + *α*_2_∑_*i*=1_^*m*^ *TC*_*i*_ + *α*_3_∑_*i*=1_^*m*^ *TADC*_*i*_ + *α*_4_∑_*i*=1_^*m*^∑_*j*=1_^*n*_*i*_^ *G*_*ij*_ *u*_*ij*_, taking the derivative by *u*_*i*[*j*]_ to (9), we have *df*(*π*, *u*)/*u*_*i*[*j*]_ = *α*_4_ *G*_*i*[*j*]_ − *β*_*i*[*j*]_ *Ω*_*ij*_ for *i* = 1,2,…, *m*; *j* = 1,2,…, *n*_*i*_. Hence, if *α*_4_ *G*_*i*[*j*]_ − *β*_*i*[*j*]_ *Ω*_*ij*_ > 0, we should not allocate any resource to job *J*_*i*[*j*]_; if *α*_4_ *G*_*i*[*j*]_ − *β*_*i*[*j*]_ *Ω*_*ij*_ < 0, we will allocate the maximal feasible amount of resource to job *J*_*i*[*j*]_; and if *α*_4_ *G*_*i*[*j*]_ − *β*_*i*[*j*]_ *Ω*_*ij*_ = 0, any feasible resource allocation can be optimal.

We define *x*_*ijr*_ = 1 if job *J*_*j*_ is scheduled in position *r* on machine *M*_*i*_, and *x*_*ijr*_ = 0 otherwise. If the number of jobs on machine *M*_*i*_ is known in advance, then we formulate the *Rm* | *p*_*ij*_ = *a*_*ij*_ *r* ^*b*_*ij*_^ + *αt* − *θ*_*ij*_ *u*_*ij*_ | *α*_1_∑_*i*=1_^*m*^ *L*_*i*_ + *α*_2_∑_*i*=1_^*m*^ *TC*_*i*_ + *α*_3_∑_*i*=1_^*m*^ *TADC*_*i*_ + *α*_4_∑_*i*=1_^*m*^∑_*j*=1_^*n*_*i*_^ *G*_*ij*_ *u*_*ij*_ problem as the following assignment problem:

(12) $$\begin{array}{c} \mathrm{Min}\;Z=\overset{m}{\underset{i=1}{\sum }}\;\overset{n_{i}}{\underset{r=1}{\sum }}\;\overset{n}{\underset{j=1}{\sum }}\lambda_{ijr}x_{ijr} \end{array}$$

subject to

(13) $$\begin{array}{c} \overset{m}{\underset{i=1}{\sum }}\;\overset{n_{i}}{\underset{r=1}{\sum }}x_{ijr}=1,\;j=1,2,\ldots ,n, \end{array}$$

(14) $$\begin{array}{c} \overset{n}{\underset{j=1}{\sum }}x_{ijr}=1,\;i=1,2,\ldots ,m;\;\;r=1,2,\ldots ,n_{i}, \end{array}$$

(15) xijr=0  or  1, i=1,2,…,m;r=1,2,…,ni; j=1,2,…,n,

where

(16) λijr={Ωiraijrbij,if  α4Gij−βijΩir≥0,Ωiraijrbij+(α4Gij−βijΩir)u−ij,if  α4Gij−βijΩir<0.

Constraint (13) makes sure that each job is scheduled exactly once. Constraint (14) makes sure that each position on each machine is taken by one job.

Next, the question is how many *P*(*n*, *m*) = (*n*_1_, *n*_2_,…, *n*_*m*_) vectors exist. Note that *n*_*i*_ may be 0,1, 2,…, *n* for *i* = 1,2,…, *m*. So, if we get the numbers of jobs on the first *m* − 1 machines, the number of jobs processed on the last machine is then determined uniquely due to *n*_1_ + *n*_2_ + ⋯+*n*_*m*_ = *n*. Therefore, the upper bound of the number of *P*(*n*, *m*) vectors is (*n* + 1)^*m*−1^. Based on the above analysis, we have the following result.

**Theorem 2**

The problem *Rm* | *p*_*ij*_ = *a*_*ij*_ *r* ^*b*_*ij*_^ + *αt* − *θ*_*ij*_ *u*_*ij*_ | *α*_1_∑_*i*=1_^*m*^ *L*_*i*_ + *α*_2_∑_*i*=1_^*m*^ *TC*_*i*_ + *α*_3_∑_*i*=1_^*m*^ *TADC*_*i*_ + *α*_4_∑_*i*=1_^*m*^∑_*j*=1_^*n*_*i*_^ *G*_*ij*_ *u*_*ij*_ can be solved in *O*(*n* ^*m*+2^) time; that is, the problem is polynomially solvable because *m* is a constant.

Base on the above analysis, we can determine the optimal solution for the problem *Rm* | *p*_*ij*_ = *a*_*ij*_ *r* ^*b*_*ij*_^ + *αt* − *θ*_*ij*_ *u*_*ij*_ | *α*_1_∑_*i*=1_^*m*^ *L*_*i*_ + *α*_2_∑_*i*=1_^*m*^ *TC*_*i*_ + *α*_3_∑_*i*=1_^*m*^ *TADC*_*i*_ + *α*_4_∑_*i*=1_^*m*^∑_*j*=1_^*n*_*i*_^ *G*_*ij*_ *u*_*ij*_ via the following algorithm.

**Algorithm 3**

Consider the following.

*Step  1*. For all the possible vectors (*n*_1_, *n*_2_,…, *n*_*m*_), solve the assignment problems ((12)–(16)). Then, obtain the optimal schedule and the corresponding total cost *α*_1_∑_*i*=1_^*m*^ *L*_*i*_ + *α*_2_∑_*i*=1_^*m*^ *TC*_*i*_ + *α*_3_∑_*i*=1_^*m*^ *TADC*_*i*_ + *α*_4_∑_*i*=1_^*m*^∑_*j*=1_^*n*_*i*_^ *G*_*ij*_ *u*_*ij*_ for each possible vector (*n*_1_, *n*_2_,…, *n*_*m*_).

*Step  2*. The optimal solution for the problem is the one with the minimum value of the total cost *α*_1_∑_*i*=1_^*m*^ *L*_*i*_ + *α*_2_∑_*i*=1_^*m*^ *TC*_*i*_ + *α*_3_∑_*i*=1_^*m*^ *TADC*_*i*_ + *α*_4_∑_*i*=1_^*m*^∑_*j*=1_^*n*_*i*_^ *G*_*ij*_ *u*_*ij*_.

*Step  3*. Calculate the optimal resources allocation by using (11).

The following example illustrates the working of Algorithm 3 for the problem *Rm* | *p*_*ij*_ = *a*_*ij*_ *r* ^*b*_*ij*_^ + *αt* − *θ*_*ij*_ *u*_*ij*_ | *α*_1_∑_*i*=1_^*m*^ *L*_*i*_ + *α*_2_∑_*i*=1_^*m*^ *TC*_*i*_ + *α*_3_∑_*i*=1_^*m*^ *TADC*_*i*_ + *α*_4_∑_*i*=1_^*m*^∑_*j*=1_^*n*_*i*_^ *G*_*ij*_ *u*_*ij*_.

**Example 4**

*Data.* *n* = 4, *m* = 2, *α* = 0.05, and *α*_1_ = *α*_2_ = *α*_3_ = *α*_4_ = 1, and the other corresponding parameters are shown in Table 1.

*Solution*. When *n*_1_ = 0, *n*_2_ = 4, *Ω*_21_ = 5.9931, *Ω*_22_ = 7.6125, *Ω*_23_ = 7.2500, *Ω*_24_ = 5, then the optimal schedule on machine *M*_2_ is [*J*_4_, *J*_1_, *J*_2_, *J*_3_] and *Z*_1_ = 155.2178.

When *n*_1_ = 1, *n*_2_ = 3, *Ω*_11_ = 2, *Ω*_21_ = 4.4600, *Ω*_22_ = 5.2000, *Ω*_23_ = 4, the optimal schedule on machine *M*_1_ is [*J*_3_] and on machine *M*_2_ is [*J*_4_, *J*_1_, *J*_2_], and *Z*_1_ = 102.7967.

When *n*_1_ = 2, *n*_2_ = 2,   *Ω*_11_ = 3.1500, *Ω*_12_ = 3, *Ω*_21_ = 3.1500, *Ω*_22_ = 3, the optimal schedule on machine *M*_1_ is [*J*_2_, *J*_3_] and on machine *M*_2_ is [*J*_4_, *J*_1_], and *Z*_1_ = 99.9928.

When *n*_1_ = 3, *n*_2_ = 1, *Ω*_11_ = 4.4600, *Ω*_12_ = 5.2000, *Ω*_13_ = 4, *Ω*_21_ = 2, the optimal schedule on machine *M*_1_ is [*J*_2_, *J*_3_, *J*_4_] and on machine *M*_2_ is [*J*_1_], and *Z*_1_ = 115.8243.

When *n*_1_ = 4, *n*_2_ = 0, *Ω*_11_ = 5.9931, *Ω*_12_ = 7.6125, *Ω*_13_ = 7.2500, *Ω*_14_ = 5, the optimal schedule on machine *M*_1_ is [*J*_4_, *J*_3_, *J*_2_, *J*_1_], and *Z*_1_ = 168.1833.

Hence, the optimal schedule on machine *M*_1_ is [*J*_2_, *J*_3_] and on machine *M*_2_ is [*J*_4_, *J*_1_], *u*_1[1]_* = 4, *u*_1[2]_* = 3, *u*_2[1]_* = 2, *u*_2[2]_* = 3 and optimal cost is *Z*_1_ = 99.9928.

## 4. Problem *Rm* | *p*_*ij*_ = *a*_*ij*_ *r* ^*b*_*ij*_^ + *αt* − *θ*_*ij*_ *u*_*ij*_ | *α*_1_∑_*i*=1_^*m*^ *L*_*i*_ + *α*_2_∑_*i*=1_^*m*^ *TW*_*i*_ + *α*_3_∑_*i*=1_^*m*^ *TADW*_*i*_ + *α*_4_∑_*i*=1_^*m*^∑_*j*=1_^*n*_*i*_^ *G*_*ij*_ *u*_*ij*_

Similar to the analysis of the problem *Rm* | *p*_*ij*_ = *a*_*ij*_ *r* ^*b*_*ij*_^ + *αt* − *θ*_*ij*_ *u*_*ij*_ | *α*_1_∑_*i*=1_^*m*^ *L*_*i*_ + *α*_2_∑_*i*=1_^*m*^ *TC*_*i*_ + *α*_3_∑_*i*=1_^*m*^ *TADC*_*i*_ + *α*_4_∑_*i*=1_^*m*^∑_*j*=1_^*n*_*i*_^ *G*_*ij*_ *u*_*ij*_ presented in the previous section, if we substitute *W*_*i*[*j*]_ = ∑_*l*=1_^*j*−1^ *p*_*i*[*l*]_, *TW*_*i*_ = ∑_*j*=1_^*n*_*i*_^ *W*_*i*[*j*]_, and *TADW*_*i*_ = ∑_*j*=1_^*n*_*i*_^ *j*(*n*_*i*_ − *j*)*p*_*i*[*j*]_ (Bagchi [36]) into (7), we have

(17) Z2(π,u)=α1∑i=1m ‍∑j=1nipi[j]+α2∑i=1m ‍∑j=1ni(ni−j)pi[j] +α3∑i=1m ‍∑j=1nij(ni−j)pi[j]+α4∑i=1m ‍∑j=1niGi[j]ui[j]=∑i=1m ∑j=1niΨijai[j]jbi[j] +∑i=1m ∑j=1ni(α4Gi[j]−βi[j]Ψij)ui[j],

where *ν*_*ij*_ = *α*_1_ + *α*_2_(*n*_*i*_ − *j*) + *α*_3_ *j*(*n*_*i*_ − *j*),

(18)   Ψi1=νi1+ανi2+α(1+α)νi3+⋯+α(1+α)ni−2νini,  Ψi2=νi2+ανi3+α(1+α)νi4+⋯+α(1+α)ni−3νini,  Ψi3=νi3+ανi4+α(1+α)νi5+⋯+α(1+α)ni−4νini,     ⋮   Ψi,ni−1=νi,ni−1+ανini,    Ψini=νini.

Applying a similar analysis in the previous section, we have the following results.

**Lemma 5**

For a given sequence, the optimal resource allocation of the problem *Rm* | *p*_*ij*_ = *a*_*ij*_ *r* ^*b*_*ij*_^ + *αt* − *θ*_*ij*_ *u*_*ij*_ | *α*_1_∑_*i*=1_^*m*^ *L*_*i*_ + *α*_2_∑_*i*=1_^*m*^ *TW*_*i*_ + *α*_3_∑_*i*=1_^*m*^ *TADW*_*i*_ + *α*_4_∑_*i*=1_^*m*^∑_*j*=1_^*n*_*i*_^ *G*_*ij*_ *u*_*ij*_ can be determined by

(19) $$\begin{array}{c} u_{i[j]}^{*}=\{\begin{array}{cc} {\overset{-}{u}}_{i[j]}, & if\;\;\alpha_{4}G_{i[j]}-\beta_{i[j]}\Psi_{ij}<0,\\ u_{i[j]}\in \left[0,{\overset{-}{u}}_{i[j]}\right], & if\;\;\alpha_{4}G_{i[j]}-\beta_{i[j]}\Psi_{ij}=0,\\ 0, & if\;\;\alpha_{4}G_{i[j]}-\beta_{i[j]}\Psi_{ij}>0, \end{array} \end{array}$$

where *u*_*i*[*j*]_*, *i* = 1,2,…, *m*; *j* = 1,2,…, *n*_*i*_ represents the optimal resource allocation of the job in position *j* on machine *M*_*i*_.

**Lemma 6**

For the problem *Rm* | *p*_*ij*_ = *a*_*ij*_ *r* ^*b*_*ij*_^ + *αt* − *θ*_*ij*_ *u*_*ij*_ | *α*_1_∑_*i*=1_^*m*^ *L*_*i*_ + *α*_2_ *TW*_*i*_ + *α*_3_ *TADW*_*i*_ + *α*_4_∑_*i*=1_^*m*^∑_*j*=1_^*n*_*i*_^ *G*_*ij*_ *u*_*ij*_, if the vector *P*(*n*, *m*) = (*n*_1_, *n*_2_,…, *n*_*m*_) is given, the optimal sequence can be determined by solving the following assignment problem:

(20)
Min
 Z=∑i=1m‍ ∑r=1ni ∑j=1nθijrxijr subject  to(13),  (14)  and  (15),

where

(21) θijr={Ψiraijrbij,if  δ4Gij−βijΨir≥0,Ωiraijrbij+(α4Gij−βijΨir)mij,if  α4Gij−βijΨir<0.

**Theorem 7**

The problem *Rm* | *p*_*ij*_ = *a*_*ij*_ *r* ^*b*_*ij*_^ + *αt* − *θ*_*ij*_ *u*_*ij*_ | *α*_1_∑_*i*=1_^*m*^ *L*_*i*_ + *α*_2_ *TW*_*i*_ + *α*_3_ *TADW*_*i*_ + *α*_4_∑_*i*=1_^*m*^∑_*j*=1_^*n*_*i*_^ *G*_*ij*_ *u*_*ij*_ can be solved in *O*(*n* ^*m*+2^) time; that is, the problem is polynomially solvable because *m* is a constant.

The optimal solution for the problem *Rm* | *p*_*ij*_ = *a*_*ij*_ *r* ^*b*_*ij*_^ + *αt* − *θ*_*ij*_ *u*_*ij*_ | *α*_1_∑_*i*=1_^*m*^ *L*_*i*_ + *α*_2_ *TW*_*i*_ + *α*_3_ *TADW*_*i*_ + *α*_4_∑_*i*=1_^*m*^∑_*j*=1_^*n*_*i*_^ *G*_*ij*_ *u*_*ij*_ can be obtained by the following algorithm.

**Algorithm 8**

Consider the following.

*Step  1*. For all the possible vectors (*n*_1_, *n*_2_,…, *n*_*m*_), solve the assignment problems ((20), (13)–(15), (21)). Then, obtain the optimal schedule and the corresponding total cost *α*_1_∑_*i*=1_^*m*^ *L*_*i*_ + *α*_2_∑_*i*=1_^*m*^ *TW*_*i*_ + *α*_3_∑_*i*=1_^*m*^ *TADW*_*i*_ + *α*_4_∑_*i*=1_^*m*^∑_*j*=1_^*n*_*i*_^ *G*_*ij*_ *u*_*ij*_ for each possible vector (*n*_1_, *n*_2_,…, *n*_*m*_).

*Step  2*. The optimal solution for the problem is the one with the minimum value of the total cost *α*_1_∑_*i*=1_^*m*^ *L*_*i*_ + *α*_2_∑_*i*=1_^*m*^ *TW*_*i*_ + *α*_3_∑_*i*=1_^*m*^ *TADW*_*i*_ + *α*_4_∑_*i*=1_^*m*^∑_*j*=1_^*n*_*i*_^ *G*_*ij*_ *u*_*ij*_.

*Step  3*. Calculate the optimal resources allocation by using (19).

The following example illustrates the working of Algorithm 8 for the problem *Rm* | *p*_*ij*_ = *a*_*ij*_ *r* ^*b*_*ij*_^ + *αt* − *θ*_*ij*_ *u*_*ij*_ | *α*_1_∑_*i*=1_^*m*^ *L*_*i*_ + *α*_2_∑_*i*=1_^*m*^ *TW*_*i*_ + *α*_3_∑_*i*=1_^*m*^ *TADW*_*i*_ + *α*_4_∑_*i*=1_^*m*^∑_*j*=1_^*n*_*i*_^ *G*_*ij*_ *u*_*ij*_.

**Example 9**

The same date in Example 4 is used.

*Solution*. When *n*_1_ = 0, *n*_2_ = 4, Ψ_21_ = 7.6676, Ψ_22_ = 7.3025, Ψ_23_ = 5.0500, Ψ_24_ = 1, then the optimal schedule on machine *M*_2_ is [*J*_4_, *J*_1_, *J*_2_, *J*_3_] and *Z*_2_ = 126.7347.

When *n*_1_ = 1, *n*_2_ = 3, Ψ_11_ = 1, Ψ_21_ = 5.2525, Ψ_22_ = 4.0500, Ψ_23_ = 1, the optimal schedule on machine *M*_1_ is [*J*_3_] and on machine *M*_2_ is [*J*_4_, *J*_1_, *J*_2_], and *Z*_2_ = 84.13484.

When *n*_1_ = 2, *n*_2_ = 2, Ψ_11_ = 3.0500, Ψ_12_ = 1, Ψ_21_ = 3.0500, Ψ_22_ = 1, the optimal schedule on machine *M*_1_ is [*J*_3_, *J*_4_] and on machine *M*_2_ is [*J*_1_, *J*_2_], and *Z*_2_ = 73.1692.

When *n*_1_ = 3, *n*_2_ = 1, Ψ_11_ = 5.2525, Ψ_12_ = 4.0500, Ψ_13_ = 1, Ψ_21_ = 1, the optimal schedule on machine *M*_1_ is [*J*_3_, *J*_4_, *J*_2_] and on machine *M*_2_ is [*J*_1_], and *Z*_2_ = 95.8810.

When *n*_1_ = 4, *n*_2_ = 0, Ψ_11_ = 7.6676, Ψ_12_ = 7.3025, Ψ_13_ = 5.0500, Ψ_14_ = 1, the optimal schedule on machine *M*_1_ is [*J*_4_, *J*_3_, *J*_2_, *J*_1_], and *Z*_2_ = 144.1351.

Hence, the optimal schedule on machine *M*_1_ is [*J*_3_, *J*_4_] and on machine *M*_2_ is [*J*_1_, *J*_2_], *u*_1[1]_* = 3, *u*_1[2]_* = 1, *u*_2[1]_* = 3, *u*_2[2]_* = 0 and optimal cost is *Z*_2_ = 73.1692.

## 5. Conclusions

In this paper, we have studied the problem of scheduling *n* jobs on *m* unrelated parallel machines with simultaneous consideration of learning effect, deteriorating jobs, and controllable processing times. We provide an *O*(*n* ^*m*+2^) time algorithm for the problems *Rm* | *p*_*ij*_ = *a*_*ij*_ *r* ^*b*_*ij*_^ + *αt* − *θ*_*ij*_ *u*_*ij*_ | *α*_1_∑_*i*=1_^*m*^ *L*_*i*_ + *α*_2_∑_*i*=1_^*m*^ *TC*_*i*_ + *α*_3_∑_*i*=1_^*m*^ *TADC*_*i*_ + *α*_4_∑_*i*=1_^*m*^∑_*j*=1_^*n*_*i*_^ *G*_*ij*_ *u*_*ij*_ and *Rm* | *p*_*ij*_ = *a*_*ij*_ *r* ^*b*_*ij*_^ + *αt* − *θ*_*ij*_ *u*_*ij*_ | *α*_1_∑_*i*=1_^*m*^ *L*_*i*_ + *α*_2_∑_*i*=1_^*m*^ *TW*_*i*_ + *α*_3_∑_*i*=1_^*m*^ *TADW*_*i*_ + *α*_4_∑_*i*=1_^*m*^∑_*j*=1_^*n*_*i*_^ *G*_*ij*_ *u*_*ij*_, respectively. The algorithms can also be easily applied to the cases *b*_*ij*_ > 0 (aging effect) and *α* < 0. Future research may focus on similar problems with more general processing time model and extend the problems to flow shop machine settings.

## Figures and Tables

**Table 1:** The date of Example 4.

*J*_*j*_	*J*_1_	*J*_2_	*J*_3_	*J*_4_
*a*_1*j*_	16	20	18	14
*a*_2*j*_	17	19	20	12
*b*_1*j*_	−0.35	−0.21	−0.25	−0.31
*b*_2*j*_	−0.23	−0.31	−0.27	−0.25
θ_1*j*_	2	3	4	5
θ_2*j*_	4	5	2	3
${\overset{-}{u}}_{1j}$	3	4	3	1
${\overset{-}{u}}_{2j}$	3	2	5	2
*G*_1*j*_	6	4	2	3
*G*_2*j*_	3	7	5	4
